# A Telangiectasia Macularis Eruptiva Perstans in a Child: A Rare Vascular Phenotype of Cutaneous Mastocytosis

**DOI:** 10.1155/crpe/8849274

**Published:** 2026-06-17

**Authors:** Bouchra Baghad, Meriem Takiddine, Farida Marnissi, Soumiya Chiheb

**Affiliations:** ^1^ Department of Dermatology and Venereology, Ibn Rochd University Hospital, Hassan II University of Casablanca, Morocco, uh2c.ac.ma; ^2^ Le Palais Center for Anatomic Pathology, Casablanca, Morocco

**Keywords:** case report, cutaneous mastocytosis, KIT mutation, pediatric dermatology, telangiectasia macularis eruptiva perstans

## Abstract

Telangiectasia macularis eruptiva perstans (TMEP) is a rare vascular phenotype of cutaneous mastocytosis predominantly reported in adults; pediatric cases are exceptional and are particularly difficult to recognize in darker skin phototypes. We report the case of a 4‐year‐old girl who presented with subtle brown macules and faint telangiectasias, in whom dermoscopy and histopathology confirmed TMEP despite a normal serum tryptase level. Symptomatic management with antihistamines and topical tacrolimus achieved a favorable clinical outcome. This case highlights the diagnostic challenges of TMEP in skin of color and underscores the need for structured long‐term surveillance.

## 1. Introduction

Mastocytosis is a rare clonal disorder characterized by the excessive proliferation and accumulation of mast cells in one or more tissues [1]. In children, the disease is almost exclusively cutaneous, and most cases fall within the spectrum of cutaneous mastocytosis (CM), which includes maculopapular CM (MPCM), diffuse CM (DCM), and solitary mastocytoma [2]. Telangiectasia macularis eruptiva perstans (TMEP) is a rare clinical phenotype within this spectrum. Indeed, according to the 2022 WHO classification [3], TMEP is now classified under MPCM, as patients with telangiectatic lesions frequently exhibit maculopapular features elsewhere. Unlike classic MPCM, TMEP is distinguished by persistent macular telangiectasias, sparse dermal mast‐cell infiltrates, and a frequently negative Darier’s sign, reflecting its predominant vascular phenotype. Although predominantly reported in adults, TMEP is exceptionally rare in children [4].

Accurate classification of mastocytosis is essential, as lesion morphology, distribution, and variant subtyping correlate with prognosis and guide follow‐up strategies. Consensus classifications, updated over the last decade, distinguish childhood‐onset variants from adult forms based on their distinct clinical behavior and high likelihood of spontaneous regression [1].

TMEP poses significant diagnostic challenges in children [5]. The subtle telangiectatic component may mimic capillary malformations, purpura‐like dermatoses, or pigmented vascular anomalies, particularly in children and in individuals with darker skin phototypes [6]. In Phototype IV and higher, telangiectasias may be clinically less apparent, while background pigmentation tends to predominate, thereby delaying recognition and histologic confirmation.

Because TMEP is extremely rare in children [7–9], establishing an accurate diagnosis requires careful clinical‐dermoscopic evaluation supported by histopathology and mast‐cell–specific immunostaining [10]. Early identification is crucial to ensure appropriate surveillance and to exclude systemic involvement, which, although uncommon in children, remains a recognized risk [11]. Herein, we report a rare case of pediatric TMEP in a Moroccan child.

## 2. Case Report

**FIGURE 1:**
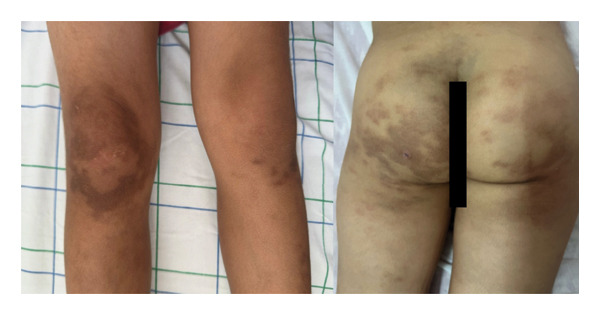
Clinical presentation of cutaneous mastocytosis (vascular variant) in a child.

A previously healthy 4‐year‐old girl (Phototype IV), with no personal or family medical history of note, presented with asymptomatic pigmented lesions of the lower limbs that had developed over a 10‐month period. Physical examination showed multiple brown macules with fine telangiectasias, symmetrically spread on the buttocks and lower extremities, especially around the knees (Figure 1). Some lesions had a bruise‐like violaceous discoloration with poorly defined borders. The macules were painless, nonindurated, and nonatrophic. Darier’s sign and dermographism were negative. There was no nail, hair, or mucosal involvement, and the patient had no systemic symptoms such as pruritus, flushing, or gastrointestinal discomfort.

**FIGURE 2:**
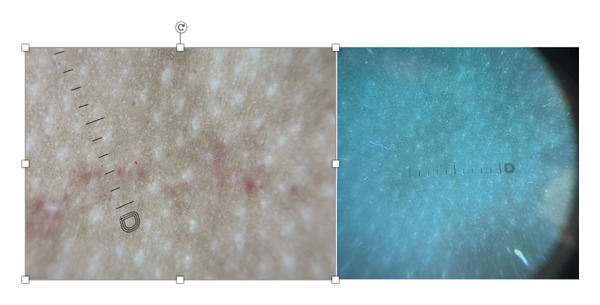
Dermoscopic features of cutaneous mastocytosis (vascular variant) in a child.

Dermoscopy demonstrated a diffuse brown background, a reticular pigmentation without a true melanocytic network, fine reticulated telangiectasias, and dotted vessels, along with whitish follicular openings and no surface scale (Figure 2). Under Wood’s lamp, the lesions exhibited a homogeneous brown–gray fluorescence without focal accentuation, suggesting a deep dermal pigmentation.

**FIGURE 3:**
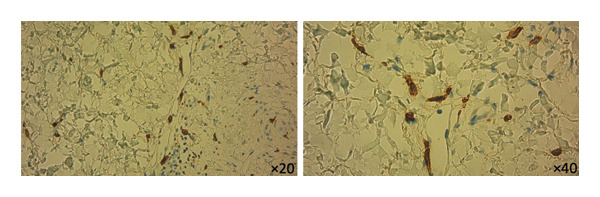
Perivascular mast‐cell infiltrate around ectatic dermal vessels in pediatric cutaneous mastocytosis.

A 4‐mm punch biopsy was obtained from a representative brown macule on the buttock. Histopathologic examination revealed a superficial perivascular mononuclear infiltrate with vascular ectasia, without features of vasculitis or lichen planus. C‐KIT (CD117) immunostaining highlighted a significant increase in perivascular and interstitial mast cells, reaching 22 mast cells per high‐power field—consistent with a mast‐cell–rich infiltrate (Figure 3). These findings, combined with the clinical and dermoscopic presentation, supported the diagnosis of TMEP.

Serum tryptase levels were normal. The patient received symptomatic treatment, including oral antihistamines and topical tacrolimus. Over a 12‐month follow‐up period, oral antihistamines achieved significant improvement of pruritus, whereas topical tacrolimus produced only slight improvement of the cutaneous lesions; the pigmented appearance of the lesions nevertheless persisted. The patient remained clinically stable throughout the follow‐up period, with no emergence of systemic symptoms. A systemic work‐up was planned only if systemic warning signs emerged. The parents were educated about disease monitoring, and a long‐term follow‐up was scheduled.

## 3. Discussion

This case illustrates a rare clinical phenotype of CM in children, characterized by an exceptionally uncommon vascular presentation, an unusual anatomical distribution, the complete absence of mast‐cell mediator–related symptoms, and a darker skin phototype in which telangiectasias are minimally apparent.

Traditionally, TMEP has been characterized as a rare vascular variant of CM, typically manifesting as diffuse, symmetrical red‐to‐brown macules with overlying telangiectasias, most often involving the trunk and proximal extremities, and occurring predominantly in adults [12]. Darier’s sign is usually negative [8, 9]. More recent data, however, suggest that TMEP may not represent a distinct disease entity but rather a highly vascularized phenotype of MPCM [13]. This shift reflects a growing understanding of mast‐cell–mediated vasodilation as the primary driver of its telangiectatic appearance.

Recognition of this clinical variant of CM may be particularly challenging in individuals with darker skin phototypes [14]. In phototypes IV–VI, the characteristic telangiectasias may be muted or clinically inapparent, as increased baseline pigmentation masks erythema and vascular patterns. Lesions may therefore manifest primarily as brown macules with subtle vascular clues, leading to potential misclassification as postinflammatory hyperpigmentation, capillary malformations, pigmented purpuric dermatoses, or dermal melanocytosis. Importantly, atypical presentations of MPCM can also mimic signs of nonaccidental trauma, making accurate recognition essential in pediatric populations [15]. Dermoscopy becomes a critical adjunct in these contexts, often demonstrating fine reticulated telangiectasias and a nonmelanocytic brown reticular pattern that strongly supports the diagnosis [16].

In the present case, several differential diagnoses were carefully considered and excluded based on clinical, dermoscopic, and histopathological findings. Postinflammatory hyperpigmentation was excluded by the absence of any preceding inflammatory or traumatic skin event and by the presence of a reticular vascular pattern on dermoscopy rather than a homogeneous brown pigmentation. Capillary malformations were ruled out based on the symmetrical and eruptive distribution of the lesions, the brownish hue rather than the typical pink–red of vascular malformations, and the absence of a congenital onset. Pigmented purpuric dermatoses were excluded clinically by the absence of petechiae or purpuric dots on dermoscopy and histologically by the absence of erythrocyte extravasation, hemosiderin deposits, or lymphocytic perivascular infiltrates typical of these conditions. Dermal melanocytosis was considered, given the violaceous coloration of some lesions; however, dermoscopy and histology did not show dendritic melanocytes or dermal melanin deposits, and CD117 immunostaining confirmed a mast‐cell–rich infiltrate rather than a melanocytic process. Finally, nonaccidental trauma was excluded by the clinical morphology (flat, nonindurated macules without ecchymotic evolution), the symmetrical distribution, and the histopathological confirmation of a mast‐cell proliferative disorder.

Histology confirms the diagnosis of CM by demonstrating perivascular/interstitial mast‐cell infiltrates, although mast‐cell thresholds remain heterogeneous in the literature. Gebhard et al. proposed a validated scoring model for the diagnosis of cutaneous lesions of mastocytosis, combining major criteria (mast cell density ≥ 27/HPF and KIT D816V mutation) with minor criteria (mast cell density ≥ 12/HPF, interstitial mast cells, mast cell clusters ≥ 3, and strong/intermediate basal pigmentation) [17]. In the present case, the mast cell density of 22 MC/HPF on CD117 staining fulfills the minor criterion of ≥ 12 MC/HPF (score = 2 points). In addition, the interstitial distribution of mast cells (score = 1 point) was documented. Applying this scoring model, the patient achieved a cumulative score of ≥ 3, placing the case within the range of “MPCM remains an unconfirmed differential diagnosis,” warranting further consideration of KIT D816V mutational analysis. However, this scoring system was developed and validated exclusively in a Caucasian population; therefore, the basal pigmentation criterion cannot be reliably applied to patients with darker skin phototypes, such as Phototype IV, in whom baseline melanin content inherently results in stronger basal pigmentation regardless of the presence of mastocytosis. This limitation underscores the need for adapted histopathological scoring systems that account for the diversity of skin phototypes. More broadly, the histopathological diagnosis of CM remains challenging, particularly in pediatric cases with sparse or interstitial mast‐cell infiltrates, and review by a dermatopathology reference center could further strengthen the diagnostic confidence in atypical or borderline presentations [5]. Serum tryptase is often normal in children with isolated cutaneous disease but remains a helpful marker for disease extent and risk stratification [2]. Management is primarily symptomatic and focused on controlling mast‐cell mediator effects. Nonsedating H1‐antihistamines are first‐line, with H2 blockers or leukotriene antagonists added if needed [2]. Topical calcineurin inhibitors, such as pimecrolimus, are preferred over topical steroids for localized lesions given their safety profile in children [18]. Narrowband UVB phototherapy demonstrates encouraging efficacy and may represent a valuable second‐line option for managing cutaneous manifestations in pediatric mastocytosis, particularly when initial treatments are unsuccessful [19].

Although most cases of childhood mastocytosis remain confined to the skin and regress spontaneously, vascular phenotypes raise additional concerns. Adult data suggest the possibility of extracutaneous symptoms or progression to systemic disease, but pediatric evidence remains scarce [5]. Current pediatric guidelines emphasize the need for structured long‐term monitoring, including clinical review, organomegaly screening, and tryptase measurement, as elevated or rising levels may signal systemic involvement [20]. Given the rarity and uncertain natural history of TMEP in children, longitudinal surveillance is essential [2].

## 4. Conclusion

This case highlights the diagnostic challenge of TMEP in children, particularly in those with darker skin in whom telangiectasias may be subtle. Dermoscopy and histopathology are key tools for avoiding misdiagnosis. Although the prognosis is usually favorable, the uncertain evolution of this rare phenotype warrants long‐term follow‐up. Heightened awareness of uncommon presentations of CM is essential to ensure timely recognition and appropriate monitoring.

## Funding

No funding was received for this manuscript.

## Ethics Statement

Informed consent was obtained in accordance with institutional and international ethical standards.

## Consent

Written informed consent for publication of clinical information and images was obtained from the patient’s legal guardians.

## Conflicts of Interest

The authors declare no conflicts of interest.

## Data Availability

The data that support the findings of this study are available on request from the corresponding author. The data are not publicly available due to privacy or ethical restrictions.

## References

[1] Leguit R. J. , Wang S. A. , George T. I. , Tzankov A. , and Orazi A. , The International Consensus Classification of Mastocytosis and Related Entities, Virchows Archiv. (January 2023) 482, no. 1, 99–112, 10.1007/s00428-022-03423-3. 36214901

[2] Giona F. , Pediatric Mastocytosis: An Update, Mediterranean Journal of Hematology and Infectious Diseases. (November 2021) 13, no. 1, 10.4084/MJHID.2021.069. PMC8577558 34804443

[3] Khoury J. D. , Solary E. , Abla O. et al., The 5th Edition of the World Health Organization Classification of Haematolymphoid Tumours: Myeloid and Histiocytic/Dendritic Neoplasms, Leukemia. (July 2022) 36, no. 7, 1703–1719, 10.1038/s41375-022-01613-1. 35732831 PMC9252913

[4] Suryawati N. and Saputra H. , Telangiectasia Macularis Eruptiva Perstans: A Case Report and Review Literature, Journal of the Turkish Academy of Dermatology. (June 2023) 17, no. 2, 54–58, 10.4274/jtad.galenos.2023.27247.

[5] Urbina F. and Benavides A. , Telangiectatic Mastocytosis: If it is not Mastocytosis, What is it? Comment on Brockow et al. Challenges in the Diagnosis of Cutaneous Mastocytosis. Diagnostics 2024, 14, 161, Diagnostics. (May 2025) 15, no. 11, 10.3390/diagnostics15111370. PMC12155401 40506942

[6] Markeson C. , Metkowski A. , Huang S. , Hsu S. , and Heath C. , Unusual Presentation of Maculopapular Cutaneous Mastocytosis in an Infant With Skin of Color, JAAD Case Reports. (August 2023) 38, 105–107, 10.1016/j.jdcr.2023.02.025. 37521199 PMC10372046

[7] Shah R. , Schwartz R. , Patel A. , and Lambert W. , A Rare Case of Telangiectasia Macularis Eruptive Perstans in a Toddler, 2022, 10.21203/rs.3.rs-1686878/v1.

[8] Tsutsui Y. , Koga M. , Koga K. , and Imafuku S. , Child Case of Linear Variant of Telangiectasia Macularis Eruptiva Perstans, The Journal of Dermatology. (December 2019) 46, no. 12, e469–e470, 10.1111/1346-8138.15086. 31531875

[9] Leonardi S. , Vitaliti G. , Praticò A. D. , and La Rosa M. , Telangiectasia Macularis Eruptive Perstans (TMEP) in Childhood: A Case Report and Literature Review, Allergologia et Immunopathologia. (September 2012) 40, no. 5, 321–323, 10.1016/j.aller.2011.05.006. 21889827

[10] Kumar S. , Jakhar D. , and Misri R. , Dermoscopy of Telangiectasia Macularis Eruptiva Perstans, Indian Dermatology Online Journal. (2020) 11, no. 1, 131–132, 10.4103/idoj.IDOJ_312_18. 32055534 PMC7001407

[11] Gurnee E. A. , Johansen M. L. , Phung T. L. et al., Pediatric Maculopapular Cutaneous Mastocytosis: Retrospective Review of Signs, Symptoms, and Associated Conditions, Pediatric Dermatology. (January 2021) 38, no. 1, 159–163, 10.1111/pde.14399. 33068315

[12] Severino M. , Chandesris M. O. , Barete S. et al., Telangiectasia Macularis Eruptiva Perstans (TMEP): A Form of Cutaneous Mastocytosis With Potential Systemic Involvement, Journal of the American Academy of Dermatology. (May 2016) 74, no. 5, 885–891.e1, 10.1016/j.jaad.2015.10.050. 26899198

[13] Wilmas K. , Wohlmuth-Wieser I. , and Duvic M. , Telangiectasia Macularis Eruptiva Perstans: An Old Terminology, Still Frequently Used, Dermatology Online Journal. (August 2017) 23, no. 8, 13030–qt6zx243zm, 10.5070/d3238036000. 29469737

[14] Walker T. D. , Varra V. , Fosu N. , Paradiso M. , and Mosser‐Goldfarb J. , Cutaneous Mastocytosis in Pediatric Patients With Skin of Color: A Retrospective Cohort Study, Pediatric Dermatology. (2025) 42, no. 4, 793–795, 10.1111/pde.15881. 39887532 PMC12285553

[15] Ovid [Internet] , Pediatric Cutaneous Mastocytosis Mistaken for, Journal of Cutaneous Medicine and Surgery. (November 2025) https://www.ovid.com/journals/jcmas/fulltext/10.1177/12034754251320636%7Epediatric-cutaneous-mastocytosis-mistaken-for-non-accidental. 10.1177/12034754251320636 39953912

[16] Sławińska M. , Kaszuba A. , Lange M. , Nowicki R. J. , Sobjanek M. , and Errichetti E. , Dermoscopic Features of Different Forms of Cutaneous Mastocytosis: A Systematic Review, Journal of Clinical Medicine. (August 2022) 11, no. 16, 10.3390/jcm11164649. PMC9410418 36012900

[17] Gebhard J. , Horny H. P. , Kristensen T. et al., Validation of Dermatopathological Criteria to Diagnose Cutaneous Lesions of Mastocytosis: Importance of KIT D816V Mutation Analysis, Journal of the European Academy of Dermatology and Venereology. (August 2022) 36, no. 8, 1367–1375, 10.1111/jdv.18143. 35412687

[18] Mashiah J. , Harel A. , Bodemer C. et al., Topical Pimecrolimus for Paediatric Cutaneous Mastocytosis, Clinical and Experimental Dermatology. (July 2018) 43, no. 5, 559–565, 10.1111/ced.13391. 29460435

[19] Brazzelli V. , Bossi G. , Bonelli A. et al., A Case of Pediatric Indolent Systemic Mastocytosis: The Role of UVB-NB Phototherapy in the Treatment of Cutaneous Lesions, Photodermatology, Photoimmunology and Photomedicine. (2023) 39, no. 5, 540–542, 10.1111/phpp.12894. 37326545

[20] Madigan L. M. , Boggs N. A. , Rets A. V. et al., Mastocytosis in the Skin: Approach to Diagnosis, Evaluation, and Management in Adult and Pediatric Patients, American Journal of Clinical Dermatology. (July 2025) 26, no. 4, 499–510, 10.1007/s40257-025-00947-7. 40392511 PMC12325572

